# Anti-Angiogenic Effect of Asperchalasine A Via Attenuation of VEGF Signaling

**DOI:** 10.3390/biom9080358

**Published:** 2019-08-12

**Authors:** Jun Yeon Park, Young Seok Ji, Hucheng Zhu, Yonghui Zhang, Do Hwi Park, Young-Joo Kim, Hye Hyun Yoo, Ki Sung Kang

**Affiliations:** 1 Department of Food Science and Biotechnology, Kyonggi University, Suwon 16227, Korea; 2 Institute of Pharmaceutical Science and Technology and College of Pharmacy, Hanyang University, Ansan 15588, Korea; 3 School of Pharmacy, Tongji Medical College of Huazhong University of Science and Technology, Wuhan 430030, China; 4 College of Korean Medicine, Gachon University, Seongnam 13120, Korea; 5 Natural Products Research Center, Korea Institute of Science and Technology, Gangneung, Gangwon-do 25451, Korea

**Keywords:** angiogenesis, metastasis, HUVEC, asperchalasine A, VEGF

## Abstract

Cytochalasans are a group of structurally diverse fungal polyketide-amino acid hybrid metabolites that exhibit diverse biological functions. Asperchalasine A was identified and isolated from an extract of the marine-derived fungus, *Aspergillus*. Asperchalasine A is a cytochalasan dimer which consists of two cytochalasan molecules connected by an epicoccine. This study investigated the potential antiangiogenic effects of *Aspergillus* extract and asperchalasine A, which significantly inhibited cell adhesion and tube formation in human umbilical vein endothelial cells (HUVECs). *Aspergillus* extract and asperchalasine A decreased the vascular endothelial growth factor (VEGF) and vascular endothelial growth factor receptor (VEGFR)-2 mRNA expression in a concentration-dependent manner. In addition, *Aspergillus* extract and asperchalasine A inhibited angiogenesis via downregulation of VEGF, p-p38, p-extracellular signal-regulated protein kinase (ERK), p-VEGFR-2, and p-Akt signaling pathways. Moreover, *Aspergillus* extract and asperchalasine A significantly inhibited the amount of blood vessel formation in fertilized chicken eggs using a chorioallantoic membrane assay. Our results provide experimental evidence of this novel biological activity of the potential antiangiogenic substances, *Aspergillus* extract, and asperchalasine A. This study also suggests that *Aspergillus* extract and its active component asperchalasine A are excellent candidates as adjuvant therapeutic substances for cancer prevention and treatment.

## 1. Introduction

All the cells of the human body need blood to supply oxygen and nutrients and to remove metabolic waste. Thus, the microvascular network extends throughout the human body to maintain the homeostatic functions and metabolic activities of the cells [1]. Angiogenesis refers to a series of processes wherein new blood vessels are created from the existing blood vessels. It is required for fetal development, menstruation, and wound healing processes under normal conditions. On the other hand, angiogenesis is also known to be necessary in various diseases, such as cancer growth and metastasis, rheumatoid arthritis, and diabetic blindness [2,3]. Angiogenesis is a very tightly regulated phenomenon that is rarely observed under normal conditions. Usually, the biological factors inducing or suppressing angiogenesis are tightly balanced. However, if the factors involved in angiogenesis are misregulated, angiogenesis continues uncontrollably, and pathologies are seen [4,5].

The vascular endothelial growth factor (VEGF) is considered an important inducer of angiogenesis in various types of tumors. As angiogenesis plays an essential role in the process of metastasis [6,7], VEGF becomes an important factor in mediating angiogenesis in the pathology of chronic disorders. In cases of diabetic retinopathy, retinal capillaries occlude and the retina becomes ischemic, resulting in the increased production of VEGF [8]. Thus, inhibition of VEGF can be targeted for the treatment of diabetic nephropathy [9]. Angiogenesis plays an important role in the abnormal growth of the synovial membrane and formation of pannus, which are important in the pathophysiology of rheumatoid arthritis; therefore, inhibition of angiogenesis in rheumatoid arthritis has also been reported as a potential therapeutic target [10]. 

Cytochalasans are a class of alkaloids with a unique structure, consisting of a perhydroisoindolone core fused with a macrocyclic ring [11]. They are fungal metabolites, and are known to bind to actin filaments and block their polymerization and elongation. Consequently, cytochalasans may alter cellular morphology, inhibit cell division, and eventually cause cell apoptosis [12]. In addition, multiple roles of cytochalasans have been reported, which include immunomodulatory [13], osteogenic [14], and nematicidal activities [15]. For these reasons, the structures and biological activities of the cytochalasan class of molecules have been extensively investigated as promising lead drug candidates. 

Asperchalasine A is the first reported cytochalasan dimer, and consists of two cytochalasan molecules connected by an epicoccine. It was isolated from the fermentation broth of *Aspergillus flavipes*. Many cytochalasans have been reported to exhibit cytotoxic activities. In addition, asperchalasine A has also been known to exhibit cytotoxicity against human cancer cells. Asperchalasine A selectively inhibits cyclin A, cyclin dependent kinase (CDK) 2, and CDK6 in cancer cells to induce significant cell cycle arrest in the G1 phase [16], suggesting the potential role of asperchalasine A as a promising anti-cancer agent and a selective cell cycle regulator. The aim of the present study was to assess the effect of *Aspergillus* extract and aspochalasine A on angiogenesis in human umbilical vein endothelial cells (HUVECs) using cell-based experiments.

## 2. Materials and Methods 

### 2.1. Chemicals and Reagents

The extract of the fermentation broth of marine-derived fungus *Aspergillus flavipes* and asperchalasine A (>99.0% purity by HPLC) were obtained from the Hubei Key Laboratory of Natural Medicinal Chemistry and Resource Evaluation, School of Pharmacy, Tongji Medical College, Huazhong University of Science and Technology, China. The preparation methods have been described previously [16]. The Clonetics EGM-2 MV BulletKit and fetal bovine serum (FBS) were purchased from Takara Bio Inc. (Shiga, Japan). An EZ-Cytox Enhanced Cell Viability Assay Kit was purchased from ITSBIO (Seoul, Korea).

### 2.2. Cell Proliferation Assay

The cytotoxicity of *Aspergillus* extract on HUVECs was assessed using an EZ-Cytox Enhanced Cell Viability Assay Kit [4]. Cells were seeded at 2 × 10^4^ cells/μL/well in 96-well plates. The cells were treated with various concentrations of asperchalasine A, or with the dimethyl sulfoxide (DMSO) vehicle control, and incubated for 24 h at 37 °C in a humidified atmosphere of 5% CO_2_ and 95% air. After 24 h of treatment, 10 μL of kit solution was added to each well, and the plates were returned to the incubator for an additional 1 h. Sample absorbance was then measured at 450 nm using a microplate reader.

### 2.3. Cell Adhesion Assay

A 96-well plate was pre-coated with Matrigel (100 μg/mL) at 4 °C for the cell adhesion assay [4]. Excess amounts of Matrigel solution was removed after 30 min of the coating period, and the plate was air-dried. The cells (2 × 10^2^ cells/μL/well) were then plated onto the Matrigel-coated plate, and media containing the samples were added. The plate was then incubated further for 30 min at 37 °C in 5% CO_2_. The cells were fixed and stained with 4% paraformaldehyde and hematoxylin after incubation. The cell adhesion was viewed using a microscope and quantified by counting the number of cells.

### 2.4. Cell Migration Assay

The migration of endothelial cells was determined using Falcon cell culture PET (polyethylene terephthalate) inserts with a pore size of 8 μm in a 24-well dish (No 353097; Falcon, Franklin Lakes, NJ, USA) [17]. The lower surface of the filters was coated with Matrigel (5 μg/mL). Cell suspension (2 × 10^3^ cells/μL/well) was added to the upper compartment of the chamber, and 600 μL of culture medium with or without test samples were added to the lower compartment, and the chambers were incubated at 37 °C. Cells which failed to migrate were removed with cotton swabs. The migrated cells on the filter were fixed with methanol and stained with Mayer’s hematoxylin and 1% Eosin Y solution (MUTO PURE CHEMICALS CO., LTD., Tokyo, Japan). Cells were photographed, counted, and the percentage of migrated cells was calculated.

### 2.5. Tube Formation Assay

Plates (96-well) were coated with 60 μL of Matrigel (10 mg/mL), which was allowed to polymerize at 37 °C. Cells were seeded at a density of 3 × 10^2^ cells/μL/well onto the Matrigel-coated plate. Clonetics EGM-2 MV BulletKit, with or without asperchalasine A, was added. The plates were then incubated at 37 °C for 24 h. After incubation, the cells were fixed with 4% paraformaldehyde, followed by staining with Mayer’s hematoxylin. Changes in cellular morphology and tubular structure formation were observed using a light microscope. The degree of tube formation was quantified by measuring the lengths of the tubes in the captured images using ImageJ [18].

### 2.6. Western Blotting Analysis

Western blotting was conducted as reported previously [19]. In brief, whole cell lysates were prepared according to the manufacturer’s instructions using radioimmunoprecipitation assay (RIPA) buffer (Cell Signaling technology, Danvers, MA, USA) containing phenylmethylsulfonyl fluoride and a protease inhibitor cocktail. Proteins (30 µg/lane) were separated by electrophoresis and blotted onto polyvinylidene fluoride transfer membranes. Bound antibodies were visualized using enhanced chemiluminescence (ECL) reagents (GE Healthcare, Hatfield, UK) and a LAS 4000 imaging system (Fujifilm, Kanagawa, Japan) [20].

### 2.7. Semi-Quantitative Eeverse-Transcriptase PCR 

The HUVECs (3 × 10^5^ cells/well) were treated with asperchalasine A overnight. After 24 h, the total RNAs were prepared using an RNeasy Mini kit (Qiagen, Hilden, Germany) according to the manufacturer’s protocol [21]. Total RNA was then reverse-transcribed to cDNA using the AccuPower CycleScript RT premix (dT18) (Bioneer, Daejeon, Korea). Specific primers (Table 1) were used to amplify the cDNA encoding of the vascular endothelial growth factor (VEGF) and vascular endothelial growth factor receptor-2 (VEGFR-2) and glyceraldehyde-3-phosphate dehydrogenase (GAPDH) genes. To perform PCR, we used the Premix Taq polymerase (Takara Bio Inc., Tokyo, Japan) and PCR conditions as follows: denaturation (95 °C for 1 min), primer-annealing (56 °C for 30 s), and elongation (72 °C for 45 s) for 40 cycles, and one cycle of extension at 72 °C for 10 min. The PCR reactions were carried out using a Biometra T gradient Thermocycler (Göttingen, Germany).

### 2.8. Chorioallantoic Membrane Assay

Fertilized chicken eggs were incubated in a humidified atmosphere at 37 °C for three days. To separate the developing chorioallantoic membrane (CAM) from the eggshell, albumin was taken out with a syringe. After incubation for 48 h, the eggshell was peeled away to expose CAM, and then the coverslips containing vehicle alone, asperchalasine A or *Aspergillus* extract, were put on the CAM. After treatment for 48 h, the formed blood vessels were photographed under a microscope.

### 2.9. Statistical Analyses

Statistical significance was determined using analysis of variance (ANOVA), followed by multiple comparison tests with a Bonferroni adjustment. A *p*-value less than 0.05 was considered statistically significant.

## 3. Results and Discussion

**Figure 1:**
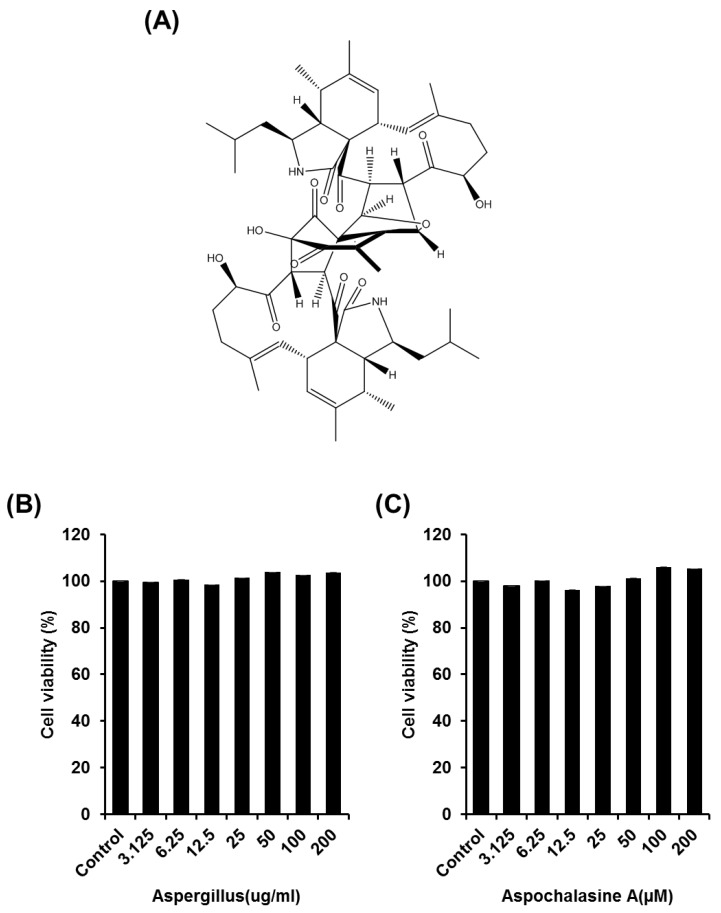
The effect of *Aspergillus* extract and asperchalasine A on human umbilical vein endothelial cells’ (HUVECs) viability. (**A**) Chemical structure of asperchalasine A. (**B**,**C**) Cells were treated with *Aspergillus* extract and asperchalasine A in a series of concentrations (3.125–200 µg/mL and 3.125–200 µM, respectively) or the dimethyl sulfoxide (DMSO) vehicle (control) for 24 h, followed by evaluation of cell viability using the EZ-Cytox assay kit. Data are expressed as mean ± standard error of the mean (SEM). Similar results were obtained in three independent experiments; * *p* < 0.05 compared to the control value.

In general, natural substances containing toxins can affect cell proliferation, growth, and cell death [22]. Therefore, assessment of the effects of natural substances on cell viability is very important. We investigated the proliferation of HUVECs, which are commonly used as in vitro angiogenesis models, to determine the effects of the *Aspergillus* extract and asperchalasine A (Figure 1) on endothelial cell growth. As shown in Figure 1, *Aspergillus* extract and asperchalasine A exhibited no significant effect on cell growth in HUVECs. As a result, we assert that *Aspergillus* extract and asperchalasine A do not cause any cytotoxicity in HUVECs.

**Figure 2:**
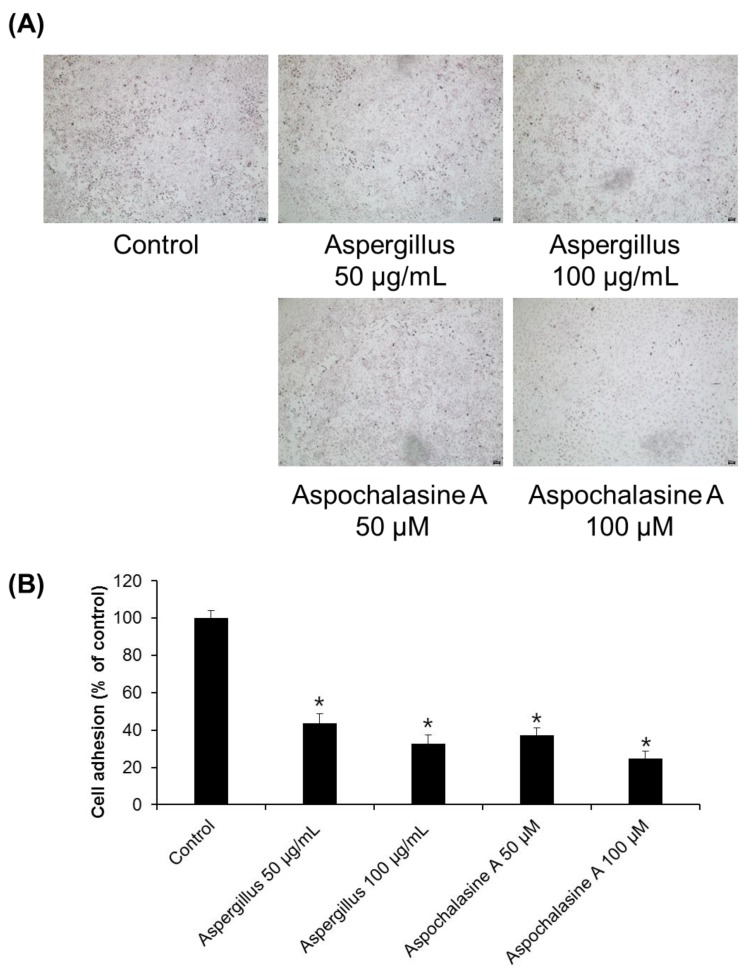
The effect of *Aspergillus* extract and aspochalasine A on HUVECs adhesion. HUVECs were treated with test samples (*Aspergillus* extract and aspochalasine A) or the DMSO vehicle (control) for 30 min. Cells that underwent adhesion were (**A**) imaged and (**B**) counted. Data are expressed as mean ± SEM. Similar results were obtained in three independent experiments; * *p* < 0.05 compared to the control value.

Angiogenesis is a complex process involving endothelial cell migration, proliferation, adhesion, tube formation, and survival [23]. Adhesion of endothelial cells to the extracellular matrix has been known to be important for endothelial cell growth, migration, differentiation, and survival [24]. The effect of the *Aspergillus* extract and asperchalasine A on cell adhesion in HUVECs was investigated using a cell adhesion assay. As shown in Figure 2, cell adhesion was significantly reduced after treatment of HUVECs. Compared with the control, 56.26 and 67.39% inhibition of cell adhesion was observed with 50 and 100 μg/mL *Aspergillus* extract, and 62.81 and 75.14% inhibition was seen with 50 and 100 μM asperchalasine A, respectively.

**Figure 3:**
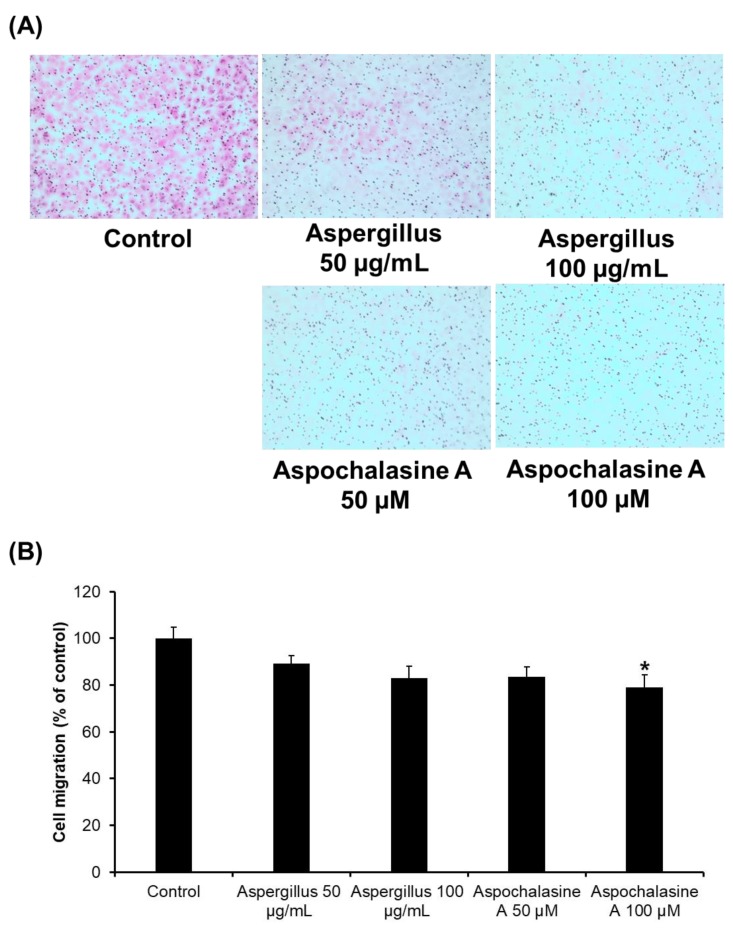
The effect of *Aspergillus* extract and aspochalasine A on HUVECs migration. (**A**) Representative images of HUVEC migration. (**B**) HUVECs were seeded into each well and treated with *Aspergillus* extract, aspochalasine A, or the DMSO vehicle (control) for 24 h. Cell migration was imaged and counted. Data are expressed as mean ± SEM. Similar results were obtained in three independent experiments; * *p* < 0.05 compared to the control value.

Directional migration of endothelial cells is an essential element of angiogenesis [25], so we investigated the effect of treatment with *Aspergillus* extract and aspochalasine A on HUVEC migration. As shown in Figure 3, the treatments with *Aspergillus* extract and aspochalasine A showed a tendency to decrease cell migration in HUVECs. Compared with the control, 10.88 and 17.06% inhibition of cell migration was observed upon treatments with 50 and 100 μg/mL *Aspergillus* extract, and 16.28 and 20.82% inhibition was noted with 50 and 100 μM aspochalasine A treatment, respectively.

**Figure 4:**
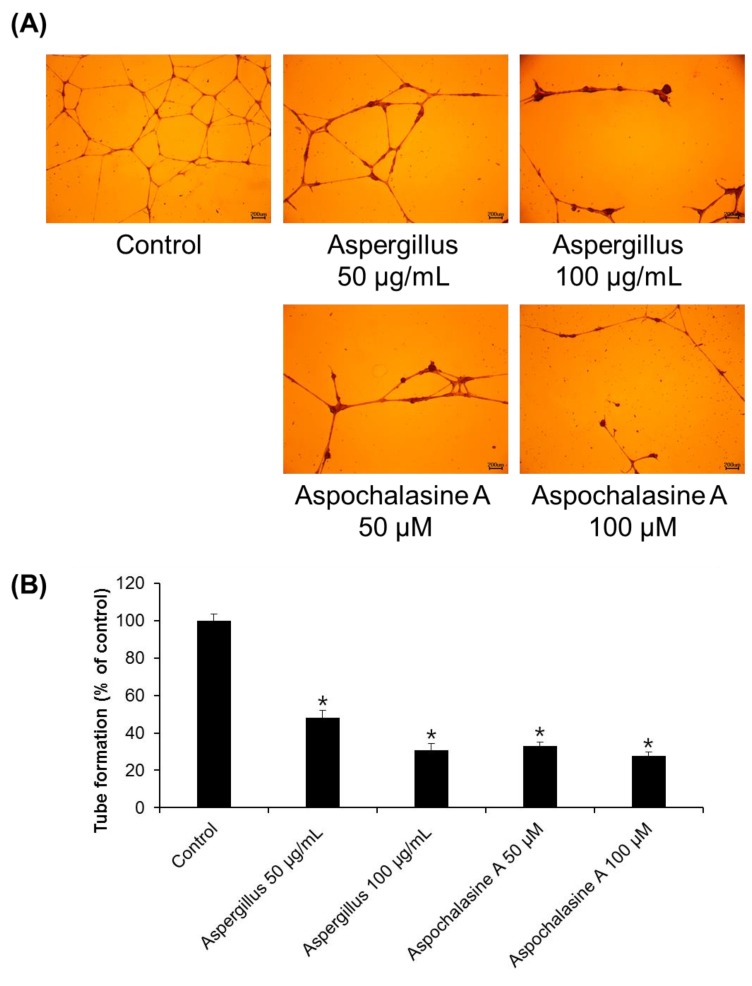
The effect of *Aspergillus* extract and aspochalasine A on HUVEC tube formation. (**A**) Representative images for tubule formation after treatment with the indicated concentrations of *Aspergillus* extract and aspochalasine A. (**B**) The length of the tubes was measured using the ImageJ software, and is represented as the percentage of tubule formation compared to the control. Data are expressed as mean ± SEM. Similar results were obtained in three independent experiments; * *p* < 0.05 compared to the control value.

Angiogenesis entails the creation of new blood vessels from existing blood vessels, and is a complex process of basement membrane dissolution, endothelial cell proliferation, and migration [26,27]. As shown in Figure 4, treatment with *Aspergillus* extract and aspochalasine A decreased tube formation in HUVECs, as was quantified by the number of branching points. Compared with the control, a 51.71 and 69.34% decrease in tube formation was achieved with 50 and 100 μg/mL *Aspergillus* extract, and a 66.98 and 72.24% decrease in tube formation was observed with 50 and 100 μM aspochalasine A treatment, respectively. These results suggest that the adhesion and migration of cells are reduced by treatment with *Aspergillus* extract and aspochalasine A, respectively, thereby inhibiting tube formation.

**Figure 5:**
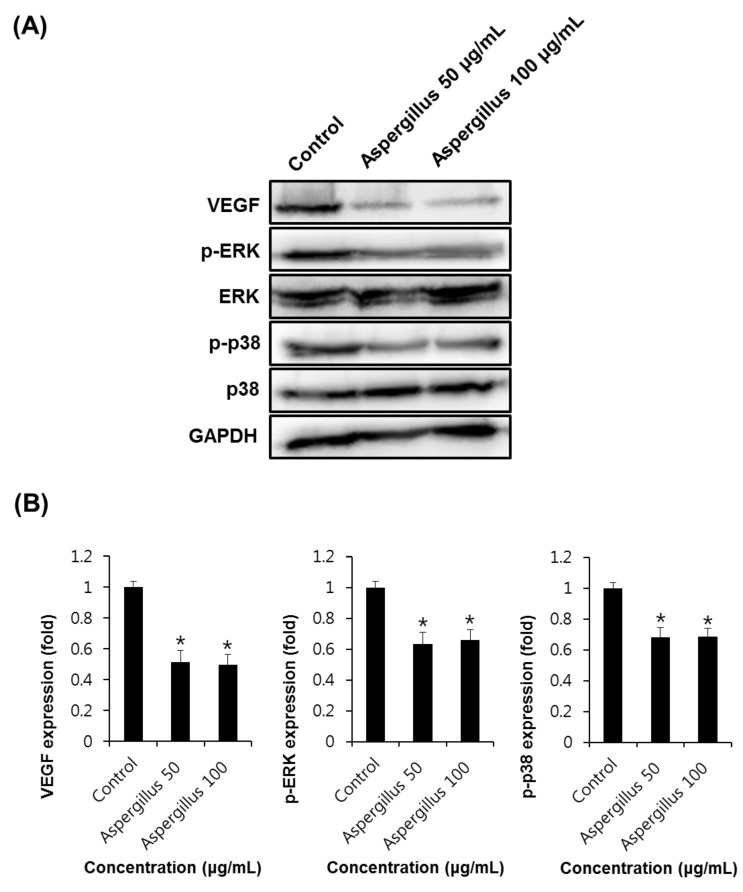
The effect of *Aspergillus* extract on angiogenic protein expression in HUVECs. **(A)** Western blot showing the levels of VEGF (21 kDa), phosphorylated ERK (42/44 kDa), ERK (42/44 kDa), phosphorylated p38 (38 kDa), and p38 (38 kDa) in HUVECs treated with *Aspergillus* extract at different concentrations for 24 h. **(B)** Graphs indicating quantification of the effect of *Aspergillus* extract on the angiogenic protein expression in HUVECs. Data are expressed as mean ± SEM. Similar results were obtained in three independent experiments; * *p* < 0.05 compared to the control value.

Angiogenesis is regulated by positive or negative effectors, such as members of the fibroblast growth factor (FGF) family, VEGF, angiogenin, and TNF-α [28]. Among these, VEGF has been reported to be the most potent regulator of angiogenesis [29]. In endothelial cells, VEGF activates downstream effectors, such as phosphoinositide-3-kinase, extracellular signal-regulated protein kinase (ERK), and AKT [30]. The phosphorylation of p38 and MAP kinase signaling pathways is known to be involved in the cell proliferation and migration of endothelial cells [31]. As shown in Figure 5, the western blot analysis indicated that the expression levels of VEGF (0.52 ± 0.02 and 0.50 ± 0.01-fold at 50 and 100 μg/mL, respectively), p-ERK (0.63 ± 0.01 and 0.66 ± 0.02-fold at 50 and 100 μg/mL, respectively), and p38 (0.68 ± 0.02 and 0.69 ± 0.01-fold at 50 and 100 μg/mL, respectively) decreased in the HUVECs treated with *Aspergillus* extract, as compared to the control.

**Figure 6:**
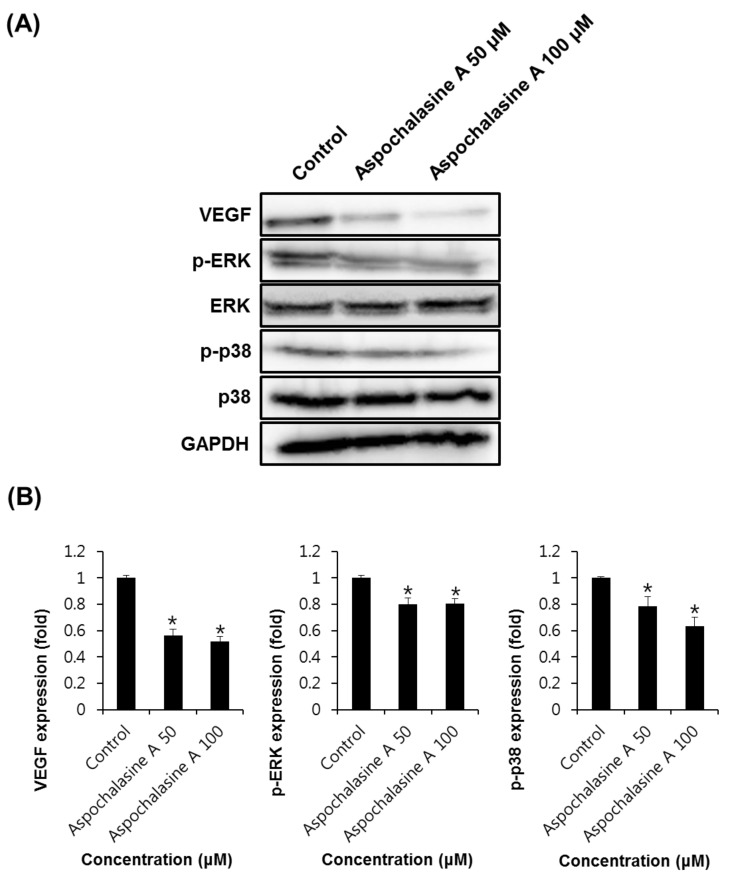
The effect of aspochalasine A on angiogenic protein expression in HUVECs. (**A**) Western blot indicating the levels of VEGF (21 kDa), phosphorylated ERK (42/44 kDa), ERK (42/44 kDa), phosphorylated p38 (38 kDa), and p38 (38 kDa) in HUVECs treated with aspochalasine A at different concentrations for 24 h. (**B**) Graphs representing quantification of effect of aspochalasine A on the angiogenic protein expressions in HUVECs. Data are expressed as mean ± SEM. Similar results were obtained in three independent experiments; * *p* < 0.05 compared to the control value.

As shown in Figure 6, western blot analysis indicated that the expression levels of VEGF (0.56 ± 0.02 and 0.52 ± 0.01-fold at 50 and 100 μM, respectively), pERK (0.80 ± 0.01 and 0.81 ± 0.02-fold at 50 and 100 μM, respectively), and p38 (0.78 ± 0.02 and 0.64 ± 0.01-fold at 50 and 100 μM, respectively) were lower in HUVECs treated with aspochalasine A than in the control. Thus, this study suggests that *Aspergillus* extract and aspochalasine A may inhibit angiogenesis by the downregulation of VEGF, phosphorylated p38, and pERK in HUVECs, and may be useful in the prevention of diseases, such as cancer.

**Figure 7:**
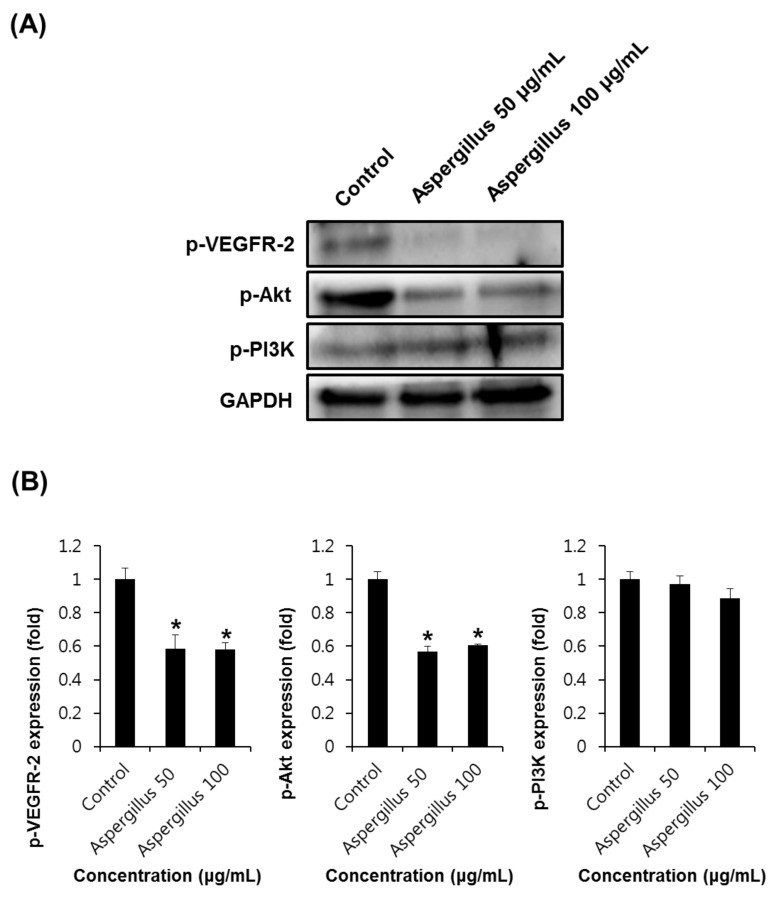

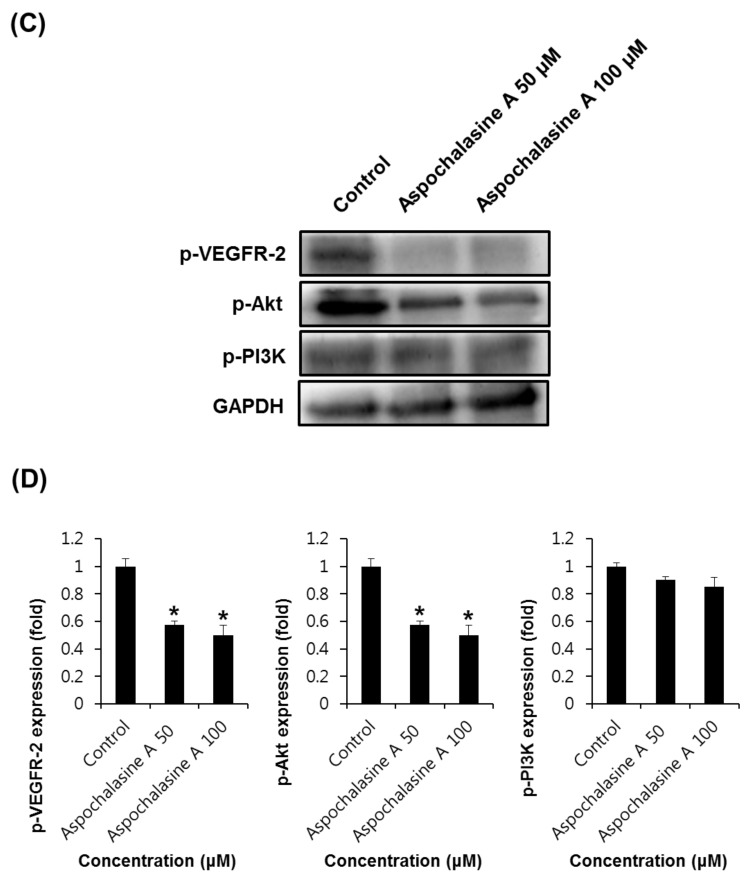
The effect of *Aspergillus* extract and aspochalasine A on VEGFR-2 and PI3K/Akt signaling in HUVECs. (**A**,**C**) Western blot indicating the levels of phosphorylated VEGFR-2 (230 kDa), VEGFR-2 (210/ 230 kDa), phosphorylated Akt (60 kDa), and phosphorylated PI3K (85 kDa) in HUVECs treated with *Aspergillus* extract and aspochalasine A at different concentrations for 24 h. (**B**,**D**) Graphs representing quantification of effect of *Aspergillus* extract and aspochalasine A on the angiogenic protein expressions in HUVECs. Data are expressed as mean ± SEM. Similar results were obtained in three independent experiments; * *p* < 0.05 compared to the control value.

VEGF and VEGFR-2 play an important role in angiogenesis, and are well-characterized signaling pathways [32]. In the process of angiogenesis, VEGF binds to the cognate receptor VEGFR-2 and results in the activation of downstream signaling molecules, including MAPK, Akt, PlC-γ, PI3K, and small GTPases [33,34]. The PI3K/Akt pathway is important for signal transduction in normal cells as well as cancer cells. This pathway plays a diverse role in angiogenesis, including cell proliferation, attachment, migration, invasion, metabolism, and survival [35,36]. Thus, VEGF/VEGFR-2 signaling activates cell survival, migration, and endothelial proliferation [37]. We investigated the effects of the *Aspergillus* extract and aspochalasine A on the phosphorylation of VEGFR-2 and PI3K/Akt. As shown in Figure 7, treatment of *Aspergillus* extract and aspochalasine A in HUVECs decreased the phosphorylation of VEGFR-2 and Akt, whereas PI3K phosphorylation was not significantly decreased by the *Aspergillus* extract and aspochalasine A in HUVECs. It is possible that decreased phosphorylation of VEGFR-2, VEGF, phosphorylation of ERK, p38, and Akt may play a sufficient role in angiogenesis.

**Figure 8:**
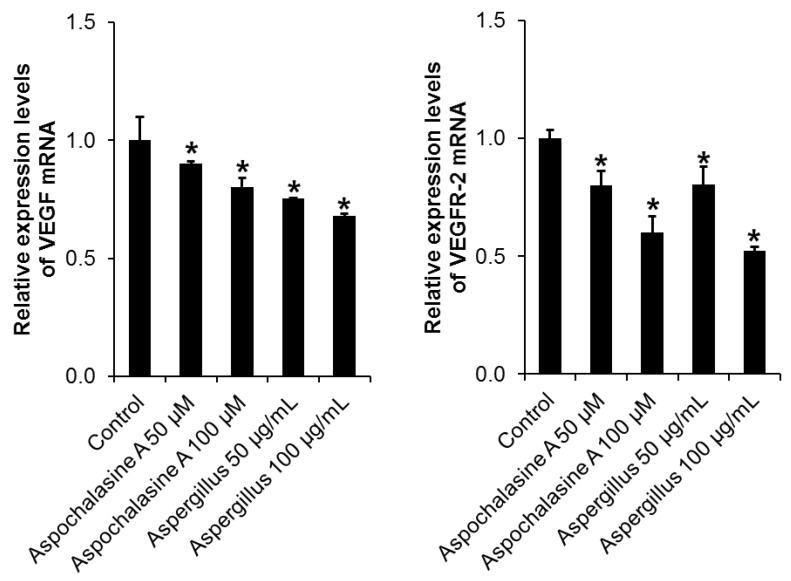
Effect of *Aspergillus* extract and aspochalasine A on VEGF and VEGFR-2 mRNA expressions in HUVECs. Total RNA was extracted from the *Aspergillus* extract and aspochalasine A-treated HUVECs, and human VEGF-A mRNA or human VEGFR-2 mRNA expression were analyzed by reverse transcriptase (RT)-PCR. GAPDH was used as an internal control. Data are expressed as means ± SEM. Similar results were obtained from three independent experiments.

We also investigated the changes in VEGF and VEGFR-2 mRNA expression when treated with *Aspergillus* extract and aspochalasine A in HUVEC. As shown in Figure 8, treatment with *Aspergillus* extract and aspochalasine A decreased VEGF and VEGFR-2 mRNA expression in a concentration-dependent manner. 

**Figure 9:**
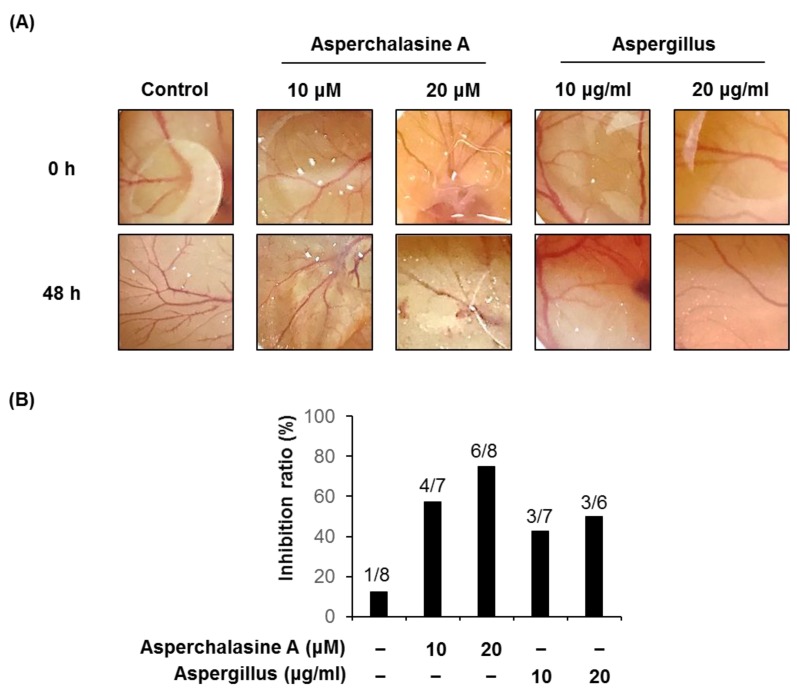
The in vivo anti-angiogenic effects of asperchalasine A and *Aspergillus* extract. (**A**) Images show representative blood vessel formation on the chorioallantoic membrane (CAM) following treatment with 10 and 20 μM asperchalasine A or 10 and 20 μg/mL *Aspergillus* extract for 48 h. (**B**) Calculations were based on the ratio of eggs with inhibited neovascularization relative to the total number of live eggs.

To verify the anti-angiogenic effects of asperchalasine A and *Aspergillus* extract in vivo, a CAM assay was carried out (Figure 9). Coverslips containing asperchalasine A (10 and 20 μM) and *Aspergillus* extract (10 and 20 μg/mL) were placed onto a CAM for 48 h, and inhibitory effects of neovascularization were observed under a microscope. Blood vessel formation in control conditions was inhibited by 12.5% at 0.5% DMSO vehicle, *n* = 8); however, treated asperchalasine A and *Aspergillus* extract significantly inhibited the number of blood vessel formation (57.1% at 10 μM asperchalasine A, *n* = 7; 75.0% at 20 μM asperchalasine A, *n* = 8; 42.9% at 10 μg/mL *Aspergillus* extract, *n* = 7; 50.0% at 20 μg/mL *Aspergillus* extract, *n* = 6). They showed no toxicity against pre-existing vessels. 

In order to overcome the disadvantages of anticancer drug therapy, such as side effects and drug resistance, many studies have been carried out using natural substances. Previous studies have shown that 7α, 15-dihydroxydehydroabietic acid isolated from *Pinus koraiensis* pinecone extract has anti-angiogenic effects [23]. Additionally, *Calvatia nipponica* extract inhibited angiogenesis through molecular mechanisms involving inhibition of VEGF/VEGFR-2, p38, MEK/ERK, and Akt signaling pathways in HUVECs [29]. However, there is a long way to go for the use of these natural products as therapeutics due to safety, bioavailability, mass synthesis, structural optimization, and clinical trials. Further studies, including animal experiments, are required for the validation of the use of these compounds.

## 4. Conclusions

In conclusion, we have confirmed the toxic effects of *Aspergillus* extract and aspochalasine A in HUVECs and the inhibitory effect on cell adhesion. Both *Aspergillus* extract and aspochalasine A inhibited tube formation through the downregulation of VEGF, p-ERK, p-p38, p-VEGFR-2, and p-Akt in HUVECs. This study suggests the possibility of the use of the *Aspergillus* extract and aspochalasine A as therapeutic agents for the inhibition of cancer metastasis.

## Figures and Tables

**Table 1:** Primer sequences used for semi-quantitative reverse-transcription polymerase chain reaction (PCR).

Gene Name	Forward Primer	Reverse Primer
*VEGF*	5′-TGCAGATTATGCGGATCAAACC-3′	5′-TGCATTCACATTTGTTGTGCTGTAG-3′
*VEGFR-2*	5′-GGAAGCTCCTGAAGATCTGT-3′	5′-GAGGATATTTCGTGCCGC-3′
*GAPDH*	5′-CAAGATTGTCAGCAACGCAT-3′	5′-GTCTTCTGGGTGGCAGTGAT-3′

## References

[1] Folkman J., Cotran R. (1976). Relation of vascular proliferation to tumor growth. Int. Rev. Exp. Pathol..

[2] Folkman J. (1995). Angiogenesis in cancer, vascular, rheumatoid and other disease. Nat. Med..

[3] Fukumura D., Gohongi T., Kadambi A., Izumi Y., Ang J., Yun C.O., Buerk D.G., Huang P.L., Jain R.K. (2001). Predominant role of endothelial nitric oxide synthase in vascular endothelial growth factor-induced angiogenesis and vascular permeability. Proc. Natl. Acad. Sci. USA.

[4] Park E.H., Park J.Y., Yoo H.S., Yoo J.E., Lee H.L. (2016). Assessment of the anti-metastatic properties of sanguiin H-6 in HUVECs and MDA-MB-231 human breast cancer cells. Bioorg. Med. Chem. Lett..

[5] Weidner N., Carroll P.R., Flax J., Blumenfeld W., Folkman J. (1993). Tumor angiogenesis correlates with metastasis in invasive prostate carcinoma. Am. J. Pathol..

[6] Carmeliet P., Ferreira V., Breier G., Pollefeyt S., Kieckens L., Gertsenstein M., Fahrig M., Vandenhoeck A., Harpal K., Eberhardt C. (1996). Abnormal blood vessel development and lethality in embryos lacking a single VEGF allele. Nature.

[7] Brown L.F., Berse B., Jackman R.W., Tognazzi K., Manseau E.J., Senger D.R., Dvorak H.F. (1993). Expression of vascular permeability factor (vascular endothelial growth factor) and its receptors in adenocarcinomas of the gastrointestinal tract. Cancer Res..

[8] Lee T.K., Park J.Y., Yu J.S., Jang T.S., Oh S.T., Pang C., Ko Y.J., Kang K.S., Kim K.H. (2018). 7α,15-Dihydroxydehydroabietic acid from *Pinus koraiensis* inhibits the promotion of angiogenesis through downregulation of VEGF, p-Akt and p-ERK in HUVECs. Bioorg. Med. Chem. Lett..

[9] Kim J.M., Ho S.H., Park E.J., Hahn W., Cho H., Jeong J.G., Lee Y.W., Kim S. (2002). Angiostatin gene transfer as an effective treatment strategy in murine collagen-induced arthritis. Arthritis Rheum..

[10] De Bandt M., Ben Mahdi M.H., Ollivier V., Grossin M., Dupuis M., Gaudry M., Bohlen P., Lipson K.E., Rice A., Wu Y. (2003). Blockade of vascular endothelial growth factor receptor I (VEGF-RI), but not VEGF-RII, suppresses joint destruction in the K/BxN model of rheumatoid arthritis. J. Immunol..

[11] Scherlach K., Boettger D., Remme N., Hertweck C. (2010). The chemistry and biology of cytochalasans. Nat. Prod. Rep..

[12] Knudsen P.B., Hanna B., Ohl S., Sellner L., Zenz T., Döhner H., Stilgenbauer S., Larsen T.O., Lichter P., Seiffert M. (2014). Chaetoglobosin A preferentially induces apoptosis in chronic lymphocytic leukemia cells by targeting the cytoskeleton. Leukemia.

[13] Hua C., Yang Y., Sun L., Dou H., Tan R., Hou Y. (2013). Chaetoglobosin F, a small molecule compound, possesses immunomodulatory properties on bone marrow-derived dendritic cells via TLR9 signaling pathway. Immunobiology.

[14] Samsonraj R.M., Paradise C.R., Dudakovic A., Sen B., Nair A.A., Dietz A.B., Deyle D.R., Cool S.M., Rubin J., van Wijnen A.J. (2018). Validation of Osteogenic Properties of Cytochalasin D by High-Resolution RNA-Sequencing in Mesenchymal Stem Cells Derived from Bone Marrow and Adipose Tissues. Stem Cells Dev..

[15] Hu Y., Zhang W., Zhang P., Ruan W., Zhu X. (2013). Nematicidal activity of chaetoglobosin A poduced by *Chaetomium globosum* NK102 against Meloidogyne incognita. J. Agric. Food Chem..

[16] Zhu H., Chen C., Xue Y., Tong Q., Li X.N., Chen X., Wang J., Yao G., Luo Z., Zhang Y. (2015). Asperchalasine A, a Cytochalasan Dimer with an Unprecedented Decacyclic Ring System, from *Aspergillus flavipes*. Angew. Chem. Int. Ed. Engl..

[17] Song H., Lee Y.J. (2017). Inhibition of hypoxia-induced cyclooxygenase-2 by Korean Red Ginseng is dependent on peroxisome proliferator-activated receptor gamma. J. Ginseng Res..

[18] Carpentier G., Martinelli M., Courty J., Cascone I. Angiogenesis analyzer for ImageJ. Proceedings of the 4th ImageJ User and Developer Conference.

[19] Lee H., Kim J., Park J.Y., Kang K.S., Park J.H., Hwang G.S. (2017). Processed *Panax ginseng*, sun ginseng, inhibits the differentiation and proliferation of 3T3-L1 preadipocytes and fat accumulation in *Caenorhabditis elegans*. J. Ginseng Res..

[20] Yoon D.H., Han C., Fang Y., Gundeti S., Lee I.-S.H., Song W.O., Hwang K.-C., Kim T.W., Sung G.-H., Park H. (2017). Inhibitory activity of *Cordyceps bassiana* extract on LPS-induced inflammation in RAW 264.7 cells by suppressing NF-κB activation. Nat. Prod. Sci..

[21] Son Y., Kim H., Yang B., Kim B., Park Y.C., Park C., Kim K. (2017). Inhibitory effects of methanol extract from *Nardostachys chinensis* on 27-hydroxycholesterol-induced differentiation of monocytic cells. Nat. Prod. Sci..

[22] Guon T., Chung H.S. (2017). Induction of apoptosis with *Moringa oleifera* fruits in HCT116 human colon cancer cells via intrinsic pathway. Nat. Prod. Sci..

[23] Norton K.-A., Popel A.S. (2016). Effects of endothelial cell proliferation and migration rates in a computational model of sprouting angiogenesis. Sci. Rep..

[24] Bischoff J. (1997). Cell adhesion and angiogenesis. J. Clin. Investig..

[25] Lamalice L., Le Boeuf F., Huot J. (2007). Endothelial cell migration during angiogenesis. Circ. Res..

[26] Park J.Y., Kwak J.H., Kang K.S., Jung E.B., Lee D.S., Lee S., Jung Y., Kim K.H., Hwang G.S., Lee H.L. (2017). Wound healing effects of deoxyshikonin isolated from Jawoongo: In vitro and in vivo studies. J. Ethnopharmacol..

[27] Oklu R., Walker T.G., Wicky S., Hesketh R. (2010). Angiogenesis and current antiangiogenic strategies for the treatment of cancer. J. Vasc. Interv. Radiol..

[28] Carmeliet P. (2000). Angiogenesis in health and disease. Nat. Med..

[29] Lee S., Park J.Y., Lee D., Seok S., Kwon Y.J., Jang T.S., Kang K.S., Kim K.H. (2017). Chemical constituents from the rare mushroom Calvatia nipponica inhibit the promotion of angiogenesis in HUVECs. Bioorg. Med. Chem. Lett..

[30] Shin M., Beane T.J., Quillien A., Male I., Zhu L.J., Lawson N.D. (2016). Vegfa signals through ERK to promote angiogenesis, but not artery differentiation. Development.

[31] Zhang Y., Liu X., Zhang J., Li L., Liu C. (2012). The expression and clinical significance of PI3K, pAkt and VEGF in colon cancer. Oncol. Lett..

[32] Kim E.J., Kwon K.A., Lee Y.E., Kim J.H., Kim S.H., Kim J.H. (2018). Korean Red Ginseng extract reduces hypoxia-induced epithelial-mesenchymal transition by repressing NF-κB and ERK1/2 pathways in colon cancer. J. Ginseng Res..

[33] Shibuya M., Claesson-Welsh L. (2006). Signal transduction by VEGF receptors in regulation of angiogenesis and lymphangiogenesis. Exp. Cell Res..

[34] Abhinand C.S., Raju R., Soumya S.J., Arya P.S., Sudhakaran P.R. (2016). VEGF-A/VEGFR2 signaling network in endothelial cells relevant to angiogenesis. J. Cell Commun. Signal..

[35] Bader A.G., Kang S., Zhao L., Vogt P.K. (2005). Oncogenic PI3K deregulates transcription and translation. Nat. Rev. Cancer..

[36] Karar J., Maity A. (2011). PI3K/AKT/mTOR Pathway in Angiogenesis. Front. Mol. Neurosci..

[37] Park J.Y., Shin M.S., Hwang G.S., Yamabe N., Yoo J.E., Kang K.S., Kim J.C., Lee J.G., Ham J., Lee H.L. (2018). Beneficial Effects of Deoxyshikonin on Delayed Wound Healing in Diabetic Mice. Int. J. Mol. Sci..

